# Sex Impacts Progression-Free Survival of Alectinib through Drug Exposure in Patients with *ALK-*Positive Non-Small Cell Lung Cancer

**DOI:** 10.34133/cancomm.0031

**Published:** 2026-05-21

**Authors:** Daan A.C. Lanser, Niels Heersche, M. Benthe Muntinghe-Wagenaar, Ma Ida Mohmaed Ali, Ezgi B. Ulas, Evert de Jonge, Esther Oomen-de Hoop, Marthe S. Paats, Idris Bahce, Sander Croes, Adrianus J. Langen, Lizza E.L. Hendriks, Anthonie J. Wekken, Alwin D.R. Huitema, Ron H.N. Schaik, Anne-Marie C. Dingemans, G.D. Marijn Veerman, Ron H.J. Mathijssen

**Affiliations:** ^1^ Department of Medical Oncology, Erasmus MC Cancer Institute, Erasmus University Medical Center, Rotterdam, Zuid-Holland, the Netherlands.; ^2^ Department of Pulmonary Medicine, Erasmus MC Cancer Institute, Erasmus University Medical Center, Rotterdam, Zuid-Holland, the Netherlands.; ^3^ Department of Clinical Chemistry, Erasmus University Medical Center, Rotterdam, Zuid-Holland, the Netherlands.; ^4^ Department of Pulmonary Medicine, University of Groningen, University Medical Center Groningen, Groningen, Groningen, the Netherlands.; ^5^ Department of Pharmacy and Pharmacology, The Netherlands Cancer Institute, Amsterdam, Noord-Holland, the Netherlands.; ^6^ Department of Pulmonary Medicine, Amsterdam University Medical Center, Amsterdam, Noord-Holland, the Netherlands.; ^7^ Department of Clinical Pharmacy & Toxicology, Maastricht University Medical Center, Cardiovascular Research Institute Maastricht, Maastricht, Limburg, the Netherlands.; ^8^ Department of Thoracic Oncology, The Netherlands Cancer Institute, Amsterdam, Noord-Holland, the Netherlands.; ^9^ Department of Pulmonary Medicine, Maastricht University Medical Center, Research Institute for Oncology and Reproduction, Maastricht, Limburg, the Netherlands.; ^10^ Princess Máxima Center for Pediatric Oncology, Utrecht, Utrecht, the Netherlands.; ^11^ Department of Clinical Pharmacy, University Medical Center Utrecht, Utrecht, Utrecht, the Netherlands.

Alectinib is a first-line treatment for metastatic non-small cell lung cancer harboring anaplastic lymphoma kinase (*ALK*) driver aberrations, occurring in 3% to 7% of patients [1]. Treatment efficacy of alectinib has repeatedly been linked to systemic exposure [2,3]. Although pharmacokinetic modeling estimated that 92% of patients should achieve adequate exposure with the starting dose in most countries (600 mg twice daily) [3], real-world data suggest that more than one-third of patients may be undertreated based on the proposed therapeutic threshold of 435 ng/mL [2]. In particular, males (46%) appear to be underdosed more frequently than females (29%) [2]. Moreover, females require dose reductions more often due to toxicity and exhibit higher alectinib trough concentrations than males at the starting dose [4]. Higher toxicity and exposure are also reported in carriers of genetic variants (i.e., peroxisome proliferator-activated receptor alpha gene [*PPARA*] 209G>A and CYP3A4*22) that reduce the expression or functionality of alectinib’s main metabolizing enzyme, cytochrome P450, isoform 3A4 (CYP3A4) [4]. Accordingly, sex and pharmacogenetic variants may also affect alectinib’s clinical efficacy based on underexposure. Therefore, we evaluated the impact of sex and *CYP3A4* genetic variants on progression-free survival (PFS) and overall survival (OS) in patients with *ALK*-positive metastatic non-small cell lung cancer.

The primary and secondary end points were PFS and OS, respectively. Medians were estimated using Kaplan–Meier, and associations with clinical variables were assessed using Cox regression. For pharmacokinetic analysis, alectinib plasma steady-state trough concentrations (C_trough,ss_) were used (Supplementary Materials). In 212 evaluable patients, including up to 2023 January 21, 120 (56.6%) were female, 16 (7.5%) were homozygous carriers of *PPARA* 209G>A, and 26 (12.3%) were carrying at least 1 CYP3A4*22 variant (Table S1). At the data cutoff on 2025 May 31, the median follow-up was 60.9 (95% confidence interval [CI]: 54.3 to 67.5) months, during which 109 patients (51.4%) experienced a PFS event, and 59 patients (27.8%) experienced an OS event.

Female patients exhibited a numerically longer median PFS compared to males (60.0 months [95% CI: 45.7 months–not reached] vs. 38.4 months [95% CI: 30.5 to 69.2 months]); however, not statistically significant (log-rank *P* = 0.140) (Fig. 1A). This was consistent with our univariable Cox regression, which yielded a hazard ratio (HR) of 1.30 for males versus females (95% CI: 0.89 to 1.90; *P* = 0.170). Moreover, no significant association was identified between PFS and *PPARA* 209G>A when comparing AA versus GA/GG patients (HR = 0.95, 95% CI: 0.48 to 1.88; *P* = 0.881) nor for CYP3A4*22 when comparing *1/*22 and *22/*22 versus *1/*1 patients (HR = 0.95, 95% CI: 0.50 to 1.77; *P* = 0.859). However, after adjustment for relevant covariates, including *ALK* treatment line, World Health Organization performance status, tumor protein p53 gene (*TP53*) status, and center of treatment, female patients showed a superior PFS compared to males (adjusted HR = 1.73, 95% CI: 1.15 to 2.59; *P* = 0.008; Table S2). For OS, female patients showed a similar, nonsignificant trend toward improved outcomes compared to males in univariable analysis (HR = 1.26, 95% CI: 0.76 to 2.10, *P* = 0.372). No effect on OS was observed for *PPARA* 209G>A and CYP3A4*22 (Table S3).

**Fig. 1.**
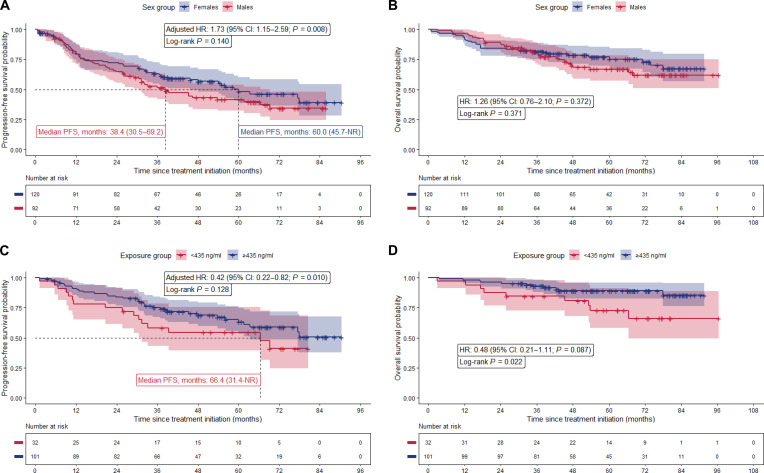
Kaplan–Meier curves for progression-free survival and overall survival in patients treated with alectinib. (A and B) Stratified by sex (males vs. females). (C and D) Stratified by exposure C_trough,ss_ threshold (≥435 ng/mL vs. <435 ng/mL). Numbers at risk are shown below the curves. CI, confidence interval; OS, overall survival; PFS, progression-free survival; *n*, number; NR; not reached; HR, hazard ratio; C_trough,ss_, trough alectinib plasma concentration.

Evaluable alectinib plasma levels were available for 133 patients (median/patient: 4; interquartile range [IQR]: 2 to 7). Median alectinib C_trough,ss_ levels were higher for females (*n* = 75; 631 ng/mL [IQR: 486 to 771 ng/mL]) than for males (*n* = 58; 466 ng/mL [IQR: 336 to 594 ng/mL; *P* < 0.001]), which was confirmed in linear regression (unadjusted β = −178, 95% CI: −249 to −106, *P* < 0.001; adjusted β = −174, 95% CI: −247 to −100, *P* < 0.001). Furthermore, a higher proportion of female patients exceeded the efficacy threshold of 435 ng/mL compared with males (89% versus 59%; *P* < 0.001), despite a higher rate of dose reductions than males (56% versus 34%; *P* = 0.001; median time of dose reduction in dose-reduced patients: 1.6 months [IQR: 0.7 to 6.4 months]). See Table S4 and Fig. S1 for the distribution of exposure to alectinib by sex.

The effect on PFS and OS of C_trough,ss_ was also evaluated in this subset of patients. It was shown that higher exposure on the log-scale was significantly associated with longer PFS in multivariable analysis (adjusted HR = 0.42, 95% CI: 0.22 to 0.82; *P* = 0.010). Using backward selection, sex was dropped as a nonsignificant variable (along with *ALK* treatment line and center of treatment), indicating that the sex effect is mediated by alectinib exposure for PFS (Table S5). A similar magnitude was observed for higher exposure and longer OS, although nonsignificantly (HR = 0.48, 95% CI: 0.21 to 1.11; *P* = 0.087; Fig. 1C and D and Table S3). Overall, males had a lower exposure, fewer dose reductions, and a shorter PFS than females. Moreover, when differentiating between dose-reduced and nondose-reduced patients, the worst outcomes were seen for nondose-reduced males (Fig. S2). Given the exposure–efficacy relationships and our observation that exposure likely mediates the sex effect, it seems plausible that males are underdosed with the flat 600-mg twice-daily starting dose.

As alectinib absorption is enhanced by food, sex-specific dietary patterns may play a role in daily practice [5]. As a previous dietary study found that females may consume more food in the morning as breakfast, their exposure might be higher due to improved absorption [6]. Unfortunately, in our study, data on dietary intake were unavailable and could not be evaluated. Another study identified body weight as a significant factor influencing alectinib clearance [3]. Considering that males had higher baseline body weight in our cohort (Table S1), this may contribute to sex-related exposure differences. However, body weight at baseline was not associated with PFS in our study. Similar sex-related differences in PFS favoring females have been observed with other kinase inhibitors, such as trametinib–dabrafenib [7], and gefitinib or erlotinib [8], although the role of drug exposure was not investigated in those cases. Finally, hormonal or immune modulation, differences in tumor biology, or sex-specific pharmacodynamics could potentially also contribute.

Although homozygous carriers of *PPARA* 209G>A showed ~30% higher exposure, absolute concentrations in both homozygous and heterozygous/wild-type patients were well above the 435 ng/mL efficacy threshold (i.e.*,* 781 versus 601 ng/mL) [4]. This variant thus leads to relative overexposure rather than underexposure and explains the lack of impact on survival. Our study is limited by the partly retrospective nature of the analysis, potentially causing selection and information bias. Since this is a multicenter analysis, the timing of data collection and local logging of clinical data could have introduced differences. For instance, missing pharmacokinetic and *TP53* status data for 37.3% and 31.1% of patients, respectively, could have introduced confounders. Also, the OS data were not mature due to the long survival of *ALK*-positive non-small cell lung cancer.

Lorlatinib appears to outperform alectinib with an impressive 5-year PFS rate of 60% [9], but direct head-to-head comparison is lacking. Nevertheless, given the potential undertreatment of male patients with alectinib, lorlatinib could potentially represent an alternative for males, especially since the observed sex-related differences do not appear to be a class effect [10].

In conclusion, this analysis demonstrates that female sex is associated with longer PFS on alectinib, with males having a 73% higher hazard of disease progression than females after adjustment. Furthermore, subset analysis suggests that this sex effect may be driven by differences in alectinib exposure between males and females, highlighting the importance of individualized dosing and therapy.

## Ethical Approval

The study protocol received primary ethical approval from the Erasmus University Medical Center (MEC 2022-158), with additional approval granted by local ethics committees at all participating centers: The Netherlands Cancer Institute (IRBdm23-071), Amsterdam University Medical Center (UVB23-0144), University Medical Center Groningen (OLS048-202211091), and Maastricht University Medical Center (2019-1080-A-11).

## Data Availability

All datasets, including deidentified participant data, which are generated during and/or analyzed during the current study, are available from the corresponding author (a.mathijssen@erasmusmc.nl) on reasonable request. Data recipients are required to enter a formal data sharing agreement that describes the conditions for release and requirements for data transfer, storage, archiving, publication, and intellectual property with each participating center.
