# Supplementary material for: Sex Impacts Progression-Free Survival of Alectinib through Drug Exposure in Patients with ALK-Positive Non-Small Cell Lung Cancer
Source: Cancer Commun (Lond). 2026 May 21;46:0031. doi: 10.34133/cancomm.0031 (PMC13191087; doi:10.34133/cancomm.0031)
Supplement: Supplementary 1 — Supplementary Text Figs. S1 and S2 Tables S1 to S5 [file cancomm.0031.f1.pdf]

Supplementary Materials for

Sex impacts progression-free survival of alectinib through drug exposure in patients with *ALK*-positive non-small cell lung cancer

Daan A.C. Lanser[1,2,3], Niels Heersche[1,3], M. Benthe Muntinghe-Wagenaar[4], Ma Ida Mohmaed Ali[5], Ezgi B. Ulas[6], Evert de Jonge[3], Esther Oomen-de Hoop[1], Marthe S. Paats[2], Idris Bahce[6], Sander Croes[7], Adrianus J. de Langen[8], Lizza E.L. Hendriks[9], Anthonie J. van der Wekken[4], Alwin D.R. Huitema[5,10,11], Ron H.N. van Schaik[3], Anne-Marie C. Dingemans[2], G.D. Marijn Veerman[2], Ron H.J. Mathijssen[1*]

Affiliations

[1] Department of Medical Oncology, Erasmus MC Cancer Institute, Erasmus University Medical Center, Rotterdam, Zuid-Holland, the Netherlands.

[2] Department of Pulmonary Medicine, Erasmus MC Cancer Institute, Erasmus University Medical Center, Rotterdam, Zuid-Holland, the Netherlands.

[3] Department of Clinical Chemistry, Erasmus University Medical Center, Rotterdam, Zuid-Holland, the Netherlands.

[4] Department of Pulmonary Medicine, University of Groningen, University Medical Center Groningen, Groningen, Groningen, the Netherlands.

[5] Department of Pharmacy and Pharmacology, The Netherlands Cancer Institute, Amsterdam, Noord-Holland, the Netherlands.

[6] Department of Pulmonary Medicine, Amsterdam University Medical Center, Amsterdam, Noord-Holland, the Netherlands.

[7] Department of Clinical Pharmacy & Toxicology, Maastricht University Medical Center, Cardiovascular Research Institute Maastricht, Maastricht, Limburg, the Netherlands.

[8] Department of Thoracic Oncology, The Netherlands Cancer Institute, Amsterdam, Noord-Holland, the Netherlands.

[9] Department of Pulmonary Medicine, Maastricht University Medical Center, Research Institute for Oncology and Reproduction, Limburg, the Netherlands.

[10] Princess Máxima Center for Pediatric Oncology, Utrecht, Utrecht, the Netherlands.

[11] Department of Clinical Pharmacy, University Medical Center Utrecht, Utrecht, Utrecht, the Netherlands.

[*]Corresponding Author

RHJM (Ron H.J. Mathijssen), a.mathijssen@erasmusmc.nl.

Material and methods

This nation-wide observational cohort study was performed in five cancer centers across the Netherlands, including the Erasmus MC Cancer Institute Rotterdam, University Medical Center Groningen, Maastricht University Medical Center, Amsterdam University Medical Center, and the Netherlands Cancer Institute. Clinical data and patient materials were collected through the following studies or initiatives: CodeGeno study (MEC 02-1002), START-TKI study (MEC 16-643; NCT05221372), Cancer Center Amsterdam – Liquid Biopsy Center (MEC 2017.333 and 2017.545), and the OncoLifeS Databiobank Groningen (https://umcgresearch.org/w/oncolifes). At the Netherlands Cancer Institute, alectinib plasma concentrations were determined routinely as part of the standard of care.

Patients with metastatic non-small cell lung cancer and a pathologically confirmed anaplastic lymphoma kinase (*ALK)* alteration who were treated with alectinib were eligible for inclusion. *ALK*-alteration had to be confirmed by immunohistochemistry or fluorescence in situ hybridization, or identified as a known echinoderm microtubule-associated protein-like 4 *(EML4)-ALK* fusion detected via next-generation sequencing or Archer FusionPlex. Prior systemic therapy—including chemotherapy, immunotherapy, or other *ALK* inhibitors—was permitted. Baseline characteristics (*e.g.*, age, body mass index), clinical data (*e.g.*, World Health Organization performance status), molecular data, and treatment duration were extracted from existing medical records. Data was collected until May 31, 2025.

Genomic DNA was extracted from plasma or whole blood and genotyped for *PPARA* 209G>A (rs4253728) and *CYP3A4*22* (rs35599367) using TaqMan-based PCR. In cases of insufficient signal, digital droplet PCR was performed for confirmation. Specific details regarding genotyping have been published elsewhere [1].

For pharmacokinetic analysis, alectinib plasma concentrations were collected during routine follow-up visits at the outpatient clinic. Samples were obtained at least one week after

treatment initiation or dose modification to ensure steady state concentrations were achieved. Plasma concentrations were extrapolated to 12 hours post-dose to estimate representative steady-state trough levels ($C_{trough,ss}$), using a previously published method [2]. Only samples collected at least 4 hours after alectinib intake were included in the analysis, as alectinib's maximum concentration is reached after 4 hours [3]. Plasma levels were collected independently of the administered dose to reflect therapeutic exposure during treatment. If multiple plasma concentrations were available for a patient, the mean of the individual measurements was used. Since pharmacokinetic data is usually skewed, the $C_{trough,ss}$ was log transformed for regression analysis.

The study protocol received primary ethical approval from the Erasmus University Medical Center (MEC 2022-158), with additional approval granted by local ethics committees at all participating centers: University Medical Center Groningen (OLS048-202211091), Maastricht University Medical Center (2019-1080-A-11), Amsterdam University Medical Center (UVB23-0144), and the Netherlands Cancer Institute (IRBdm23-071). This study was conducted in accordance with the latest version of the Declaration of Helsinki [4].

Study endpoints and statistical analysis

The primary endpoint was progression-free survival (PFS), defined as the time from the start of alectinib treatment to the first occurrence of either disease progression or death. Disease progression was determined based on the appearance of new lesions or tumor growth (RECIST v1.1) [5], or on clinical progression in the absence of RECIST-measurable disease (*e.g.*, pleural effusion). Overall survival (OS) was defined as the time from alectinib start until death from any cause. PFS and OS were evaluated using the Kaplan-Meier method, and associations with clinical variables were assessed through Cox proportional hazards regression analysis. After checking for co-linearity and whether the proportional hazards assumption was met using a

log-log Kaplan Meier plot and the Schoenfeld residuals test, variables with a predefined $P \leq 0.20$ in the univariable model were selected for multivariate Cox regression. Afterward, the best model was evaluated using the stepwise backward selection method. Median follow-up time was calculated using the reverse Kaplan-Meier method. Individual mean $C_{trough,ss}$ alectinib plasma levels were compared between sexes using the Mann–Whitney U-test and are reported as medians, as pharmacokinetic data are typically non-normally distributed. To evaluate the mediation effect of sex through C_{trough}, a linear regression analysis was conducted to assess both the univariable and multivariable effects of sex on the median trough alectinib concentrations.

All statistical tests were performed, and figures were made using SPSS for Windows (IBM, Armonk, NY, USA; version 28.0.1.0) and RStudio version 2025.05.1+513. $P < 0.05$ was considered significant.

References

1. Heersche, N., et al., *Sex and Common Germline Variants Affect the Toxicity Profile and Pharmacokinetics of Alectinib: A Nationwide Cohort Study in Patients With ALK-Positive NSCLC.* J Thorac Oncol, 2025. **20**(4): p. 475-486.
2. van Eerden, R.A.G., et al., *Feasibility of Extrapolating Randomly Taken Plasma Samples to Trough Levels for Therapeutic Drug Monitoring Purposes of Small Molecule Kinase Inhibitors.* Pharmaceuticals (Basel), 2021. **14**(2).
3. *Clinical Pharmacology and Biopharmaceutics Review Alectinib.* U.S. Food and Drug Administration.
4. World Medical, A., *World Medical Association Declaration of Helsinki: Ethical Principles for Medical Research Involving Human Participants.* JAMA, 2025. **333**(1): p. 71-74.
5. Eisenhauer, E.A., et al., *New response evaluation criteria in solid tumours: revised RECIST guideline (version 1.1).* Eur J Cancer, 2009. **45**(2): p. 228-247.

Supplementary Table S1. Baseline demographic and clinical characteristics by sex of patients with *ALK*-positive NSCLC treated with alectinib

Characteristics	Overall (n = 212)	Female [n = 120 (56.6%)]	Male [n = 92 (43.3%)]	*P* value [a]
**Age (year), median [IQR]**	62.0 [52.0 – 68.0]	62.2 [53.0 – 68.1]	61.5 [52.0 - 68.0]	0.866
**BMI (kg/m^2), median [IQR]**	25.8 [22.6 – 28.5]	25.8 [22.4 – 29.0]	26.0 [22.9 – 28.2]	0.845
**Body weight (kg), median [IQR]**	76.0 [65.0–86.7]	71.3 [62.0 – 80.0]	82.0 [75.0 – 93.0]	0.001
**Race or ethnic group**, *n* **(%)**				0.495
White	182 (85.8)	106 (88.3)	76 (82.6)	
All other groups	30 (14.2)	14 (11.7)	16 (17.4)	
**WHO performance status,** ***n*** **(%)**				0.956
0	91 (42.9)	51 (42.5)	40 (43.5)	
1	95 (44.8)	53 (44.2)	42 (45.7)	
2	20 (9.4)	12 (10.0)	8 (8.7)	
3	6 (2.8)	4 (3.3)	2 (2.2)	
**Smoking status,** ***n*** **(%)**				0.398
Never	118 (55.7)	69 (57.5)	49 (53.3)	
Former	74 (34.9)	45 (37.5)	29 (31.5)	
Current	13 (6.1)	4 (3.3)	9 (9.8)	
Unknown	7 (3.3)	2 (1.7)	5 (5.4)	
ALK **treatment line,** ***n*** **(%)**				0.998
1st	156 (73.6)	89 (74.2)	67 (72.8)	
≥2nd	56 (26.4)	31 (25.8)	25 (27.1)	
**Stage of disease,** ***n*** **(%)**[b]				0.211
III	14 (6.6)	11 (9.2)	3 (3.3)	
IV	194 (91.5)	105 (87.5)	89 (96.7)	
Unknown	4 (1.9)	4 (3.3)	0 (0.0)	
**Histological subtype,** ***n*** **(%)**				0.842
Adenocarcinoma	208 (98.1)	118 (98.3)	90 (97.8)	
LC-NEC	2 (0.9)	1 (0.8)	1 (1.1)	
Adeno-squamous carcinoma (mixed)	1 (0.5)	1 (0.8)	0 (0.0)	
LC-NOS	1 (0.5)	0 (0.0)	1 (1.1)	
TP53 **mutation status,** ***n*** **(%)**				0.459
Positive	26 (12.3)	16 (13.3)	10 (10.9)	
Wildtype	120 (56.6)	73 (60.8)	47 (51.1)	

Unknown	66 (31.1)	31 (25.8)	35 (38.0)	
**CNS metastasis**, ***n*** **(%)**				0.599
Yes	63 (29.7)	39 (32.5)	24 (26.1)	
No/Unknown	149 (70.3)	81 (67.5)	68 (73.9)	
**Center of treatment**, ***n*** **(%)**				0.995
Amsterdam UMC	14 (6.6)	6 (5.0)	8 (8.7)	
Erasmus MC	66 (31.1)	37 (30.8)	29 (31.5)	
Netherlands Cancer Institute	61 (28.8)	36 (30.0)	25 (27.2)	
UMC Groningen	44 (20.8)	26 (21.7)	18 (19.6)	
Maastricht UMC	27 (12.7)	15 (12.5)	12 (13.0)	
PPARA **209G>A**, ***n*** **(%)**				>0.999
AA	16 (7.5)	9 (7.5)	7 (7.6)	
GA/GG	193 (91.0)	109 (90.8)	84 (91.3)	
Unknown	3 (1.4)	2 (1.7)	1 (1.1)	
CYP3A4*22, ***n*** **(%)**				0.973
****1*22/*22*22****	26 (12.3)	14 (11.7)	12 (13.0)	
****1/*1****	167 (78.7)	94 (78.3)	73 (79.3)	
Unknown	19 (9.0)	12 (10.0)	7 (7.6)	

[a] Differences in sex were tested using a two-sided Pearson chi-square test or Fisher's exact test (if cell counts were <5) for categorical covariates, and an independent samples *t*-test for continuous covariates.
[b] Based on TNM classification of malignant tumors, 8th edition.
Abbreviations: *n*, number; IQR, interquartile range; BMI, body mass index; kg/m^2, kilogram per square meter; WHO, World Health Organization; *ALK*, anaplastic lymphoma kinase; LC-NEC, large-cell neuroendocrine carcinoma; LC-NOS, large-cell not other specified; *TP53*, tumor protein p53; CNS, central nervous system; MC, medical center; UMC, university medical center *PPARA*, peroxisome proliferator-activated receptor alpha; *CYP3A4,* cytochrome P450 isoform 3A4.

Supplementary Table S2. Cox regression analysis for progression-free survival in patients treated with alectinib (*n* = 212)

Variables	Univariable HR (95% CI; *P* value)	Multivariable HR (after backward selection; 95% CI; *P* value)
**Sex**<br>Male *vs.* female	1.30 (0.89 – 1.90; *P* = 0.170)	**1.73 (1.15 – 2.59; *P* = 0.008)**
***PPARA* 209G>A**<br>*AA* (*n* = 16) *vs.*<br>*GA/GG* (*n* = 193)	0.95 (0.48 – 1.88; *P* = 0.881)	NA[a]
CYP3A4*22<br>**1*22/*22*22* (*n* = 26) *vs.*<br>**1/*1* (*n* = 167)	0.95 (0.50 – 1.77; *P* = 0.859)	NA[a]
***ALK* treatment line**<br>≥2nd *vs.* 1st	**1.57 (1.05 – 2.33; *P* = 0.026)**	**1.84 (1.18 – 2.89; *P* = 0.008)**
**WHO performance status**<br>2-3 *vs.* 0-1	**2.66 (1.63 – 4.35; *P* < 0.001)**	**3.30 (1.91 – 5.69; *P* < 0.001)**
***TP53* mutation status**	Global *P* = 0.190	**Global *P* = 0.002**
Positive *vs.* wildtype	**1.93 (1.14 – 3.27; *P* = 0.015)**	**2.53 (1.37 – 4.64; *P* = 0.003)**
Unknown *vs.* wildtype	0.86 (0.56 – 1.33; *P* = 0.495)	0.69 (0.39 – 1.15; *P* = 0.148)
**Center of treatment**[b]	**Global *P* < 0.001**	**Global *P* < 0.001**
Center 1 *vs.* center 2	0.61 (0.35 – 1.06; *P* = 0.078)	0.85 (0.47 – 1.55; *P* = 0.597)
Center 1 *vs.* center 3	**2.33 (1.22 – 4.44; *P* = 0.010)**	**2.68 (1.33 – 5.43; *P* = 0.006)**
Center 1 *vs.* center 4	0.79 (0.41 – 1.54; *P* = 0.495)	1.35 (0.59 – 3.09; *P* = 0.473)
Center 1 *vs.* center 5	**2.11 (1.29 – 3.46; *P* = 0.003)**	**3.09 (1.77 – 5.34; *P* < 0.001)**
**CNS metastasis on baseline**<br>Yes *vs.* no/unknown	1.30 (0.87 – 1.94; *P* = 0.200)	NA[a]
**Smoking**<br>Former/current *vs.* never	0.76 (0.51 – 1.12; *P* = 0.161)	NA[a]
**Body weight (kg)**<br>Per kg increase	1.00 (0.99 – 1.01; *P* = 0.789)	NA[a]
**Stage of disease**<br>IV *vs.* III	2.15 (0.68-6.78; *P* = 0.194)	NA[a]

[a] Not applicable as the variable either did not reach the predefined *P*-value of *P* ≤ 0.20 or was deleted after backward selection.
[b] The treatment centers where patients received alectinib. Center 1, Erasmus MC; Center 2, Netherlands Cancer Institute; Center 3, Amsterdam UMC; Center 4, Maastricht UMC; Center 5, UMC Groningen.
Abbreviations: *n*, number; HR, hazard ratio; %, percentage; CI, confidence interval; *PPARA*, peroxisome proliferator-activated receptor alpha; vs., versus; NA, not applicable; *ALK,* Anaplastic Lymphoma Kinase; WHO, World Health Organization; CNS, Central Nervous System; kg, kilogram.

Supplementary Table S3. Cox regression analysis for overall survival in patients treated with alectinib

Variables	Univariable HR (95% CI; *P* value)
**Sex**<br>Male *vs.* female (*n* = 212)	1.26 (0.76–2.10; *P* = 0.372)
***PPARA* 209G>A**<br>*AA* (*n* = 16) *vs. GA/GG* (*n* = 193)	0.77 (0.28-2.13; *P* = 0.613)
CYP3A4*22<br>**1*22/*22*22* (*n* = 26) *vs. *1/*1* (*n* = 167)	1.18 (0.53-2.64; *P* = 0.680)
**$C_{trough,ss}$ (log) [ng/mL]**<br>Per log-ng/mL increase (*n* = 133)	0.48 (0.21-1.11; *P* = 0.087)

Abbreviations: *n*, number; HR, hazard ratio; %, percentage; CI, confidence interval; *PPARA*, peroxisome proliferator-activated; *CYP3A4,* cytochrome P450 isoform 3A4receptor alpha; vs., versus; $C_{trough,ss}$, alectinib plasma steady state trough concentration.

Supplementary Table S4. Pharmacokinetic data of alectinib exposure (*n* = 133)

	Median $C_{trough,ss}$ [IQR]	Unadjusted linear regression β-coefficient	Adjusted linear regression β-coefficient[a]	Exceeding efficacy threshold (435 ng/mL)
**Females** (*n* = 75)	631 ng/mL [486-771]	Ref.	Ref.	89% [*n* = 67]
**Males** (*n* = 58)	466 ng/mL [336-594]	-178 (95% CI: -249 to -106)	-174 (95% CI: -247 to -100)	59% [*n* =34]
***P* value**	$P < 0.001$	$P < 0.001$	$P < 0.001$	$P < 0.001$

[a] Adjusted for the center of treatment, WHO performance status, *TP53* status, and *ALK* treatment line.
Abbreviations: *n*, number; %, percentage; CI, confidence interval; $C_{trough,ss}$, alectinib plasma steady state trough concentration.

Supplementary Table S5. Cox regression analysis for progression-free survival in patients with alectinib exposure data (*n* = 133)

Variables	Multivariable HR (before backward selection; 95% CI; *P* value)	Multivariable HR (after backward selection; 95% CI; *P* value)
**$C_{trough,ss}$ (log) [ng/mL]**<br>Per log-ng/mL increase	0.50 (0.23 – 1.06; *P* = 0.071)	**0.42 (0.22–0.82; *P* = 0.010)**
**Sex**<br>Male *vs.* female	1.22 (0.63 – 2.36; *P* = 0.561)	NA[a]
***ALK* treatment line**<br>≥2nd *vs.* 1st	0.85 (0.40 – 1.79; *P* = 0.667)	NA[a]
**WHO performance score**<br>2-3 *vs.* 0-1	**5.09 (2.21 – 11.77; *P* < 0.001)**	**4.43 (2.00 – 9.84; *P* < 0.001)**
***TP53* mutation status**	**Global *P* = 0.003**	**Global *P* = 0.005**
Positive *vs.* wildtype	**2.80 (1.32 – 5.93; *P* = 0.007)**	**2.40 (1.21 – 4.76; *P* = 0.012)**
Unknown *vs.* wildtype	0.58 (0.27 – 1.23; *P* = 0.152)	0.70 (0.36 – 1.37; *P* = 0.299)
**Center of treatment**[b]	Global *P* = 0.289	NA[a]
Center 1 *vs.* center 2	0.94 (0.49 – 1.81; *P* = 0.850)	NA[a]
Center 1 *vs.* center 3	NA[c]	NA[c]
Center 1 *vs.* center 4	1.80 (0.74 – 4.37; *P* = 0.197)	NA[a]
Center 1 *vs.* center 5	NA[c]	NA[c]

[a] Not applicable as the variable was deleted after backward selection.
[b] The treatment centers where patients received alectinib. Center 1, Erasmus MC; Center 2, Netherlands Cancer Institute; Center 3, Amsterdam UMC; Center 4, Maastricht UMC; Center 5, UMC Groningen.
[c] Not applicable as the patients in the specific center were not sampled for pharmacokinetics.
Abbreviations: *n*, number; HR, hazard ratio; %, percentage; CI, confidence interval; WHO, World Health Organization; *vs*., versus; NA, not applicable.

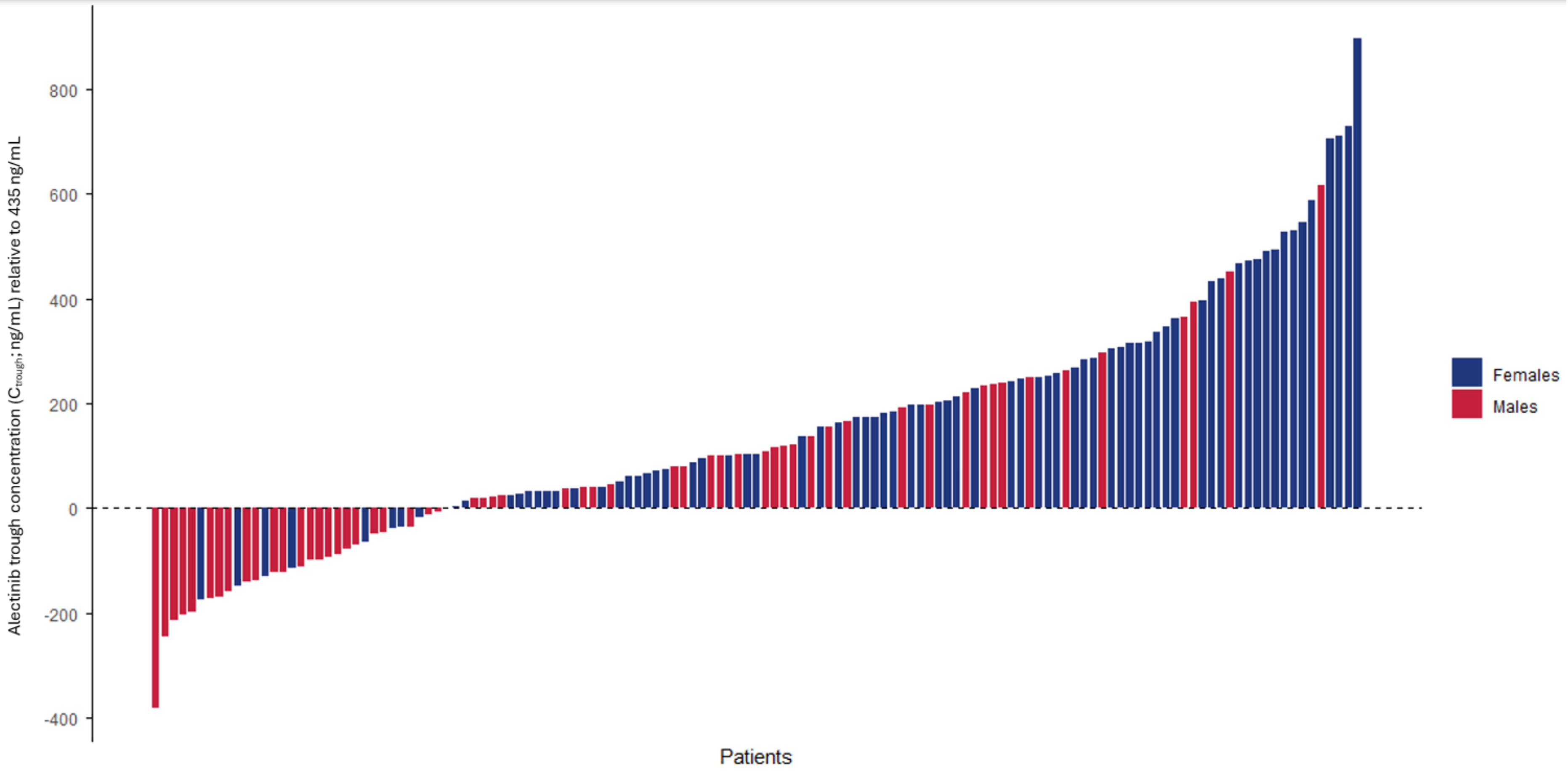

Supplementary Figure S1. Distribution of mean $C_{trough,ss}$ of alectinib for females and males (*n* = 133). Individual mean alectinib steady state trough plasma concentrations relative to the threshold of 435 ng/mL during treatment for both females and males.

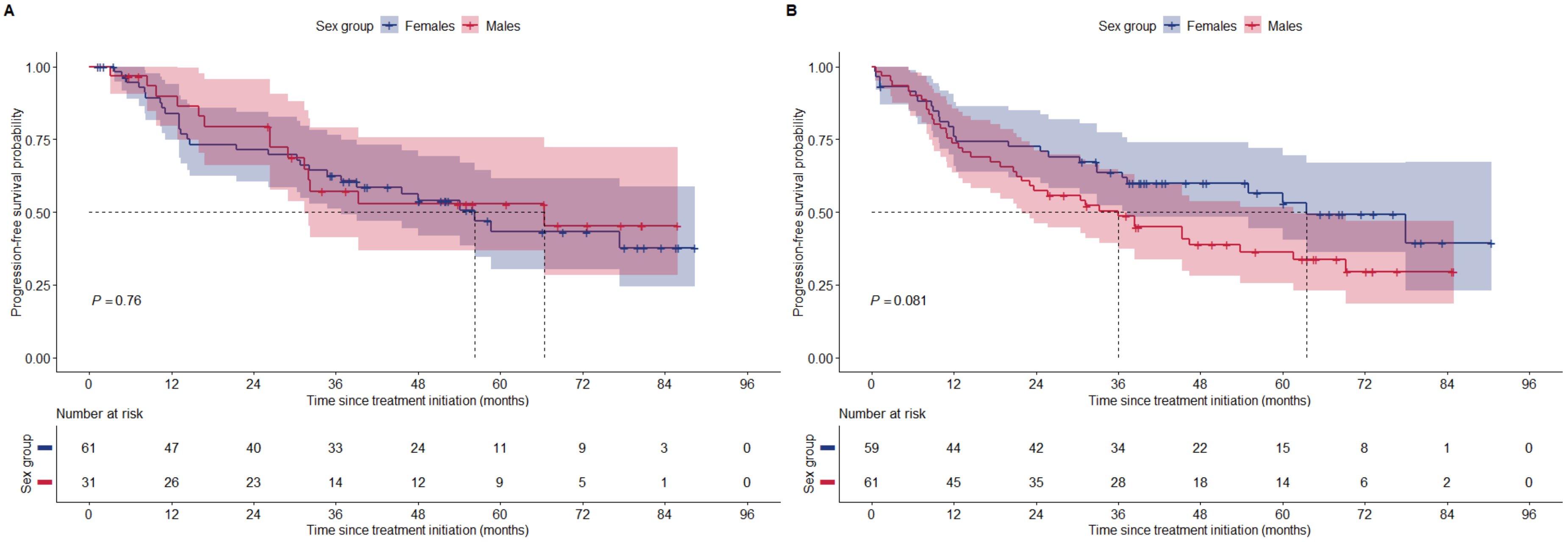

Supplementary Figure S2. Kaplan-Meier for progression-free survival between females and males, differentiating between dose-reduced and non-dose-reduced patients (*n* = 212). (A-B) Progression-free survival curves for males and females in the group with a dose reduction during the treatment course (A) and in the group without any dose reduction during the treatment course (B).
